# Inequalities in the Access to Health Services Among Older Migrants: Evidence From the China Migrant Dynamic Monitoring Survey

**DOI:** 10.3389/ijph.2023.1605325

**Published:** 2023-04-07

**Authors:** Chengxu Long, Fangfei Chen, Yisheng Ye, Lu Ji, Xinyin Xu, Shangfeng Tang

**Affiliations:** ^1^ School of Medicine and Health Management, Tongji Medical College, Huazhong University of Science and Technology, Wuhan, China; ^2^ Department of Global Health and Social Medicine, School of Global Affairs, Faculty of Social Science and Public Policy, King’s College London, London, United Kingdom; ^3^ Department of Health Service and Population Research, Institute of Psychiatry, Psychology and Neuroscience, King’s College London, London, United Kingdom; ^4^ Department of Chronic Noncommunicable Disease Control and Prevention, Sichuan Center for Disease Control and Prevention, Chengdu, China

**Keywords:** rural-to-urban migrants, older migrant workers, access to health services, social health insurance, health equity

## Abstract

**Objectives:** To identify differences in healthcare use between older migrant workers (OMWs) and older migrants (OMs) and explore associated factors and paths of healthcare use.

**Methods:** The data came from the 2015 China Migrant Dynamic Monitoring Survey (CMDMS). CMDMS used a multi-stage stratified probability proportionate to size method as the sampling technique and conducted a desk review. The samples include OMWs, OMs for caring offspring (N = 4,439), and OMs for receiving care from family (N = 4,184). We built logistic regression and path analysis models to analyze the data.

**Results:** Social health insurance (SHI) in current place of residence is associated with less expenditure among all subgroups. OMWs and OMs for receiving care from family with SHI in current place of residence are more likely to use healthcare.

**Conclusion:** OMWs are particularly vulnerable in healthcare use and socioeconomic status. Having SHI registered in current place of residence helps decrease expenditure among OMs. We urge policymakers to consider a united health financing scheme across OMWs and other urban employees and streamline policies for migrants to enroll in SHI in current place of residence.

## Introduction

The global community has been concerned with the rising prevalence of migrants and population aging. Migrants form a growing proportion of the population in many countries. The economic, social, and cultural differences that exist in the gap between the urban area and rural area inspire rural-to-urban migrants to transfer from agriculture to industry (1). In low- and middle-income countries (LMICs), rural surplus labor’s migration to cities has been fuelled by the rapid progress of urbanization (2). Rapid aging is expected to put new pressure on the migrant issue. Although older migrants (OMs) have worse health status and higher health needs, due to low socioeconomic status and existing policies which create barriers to accessing public welfare benefits, older rural-to-urban migrants are particularly vulnerable to healthcare access and at an increased risk of insufficient receipt of health services (3).

As time goes by, rural-to-urban migrant Chinese workers, a crucial portion of labor, are facing the stage of aging and have transited to a special labor group in the last 2 decades, namely, older migrant workers (OMWs). In China, the coverage and benefits of health insurance programs are not varied by the subgroups of OMs but vary across rural and urban areas (4). Due to low socioeconomic status and poor working conditions, OMWs may be at a higher risk of unmet health needs (5). Urban health financing schemes provide higher reimbursement rates than the rural health insurance scheme (6). Health and the availability of health services are highly variable between rural-to-urban migrants and urban employees (7). Although the health services coverage within health financing models was extended in the 2019 medical reform (8), the gap between rural and urban health insurance schemes still exists: the co-payment for Urban Employee Insurance (UEI) beneficiaries (24.4%) was still lower than the Urban-Rural Resident Medical Insurance (URRMI) which covers rural and urban residents without formal employment (40.3%) (9). A large number of rural-to-urban migrants usually register in their place of origin rather than their current place of residence, who are covered by URRMI or the rural health insurance scheme and excluded from UEI (10); this may be even worse for OMWs. Chinese OMWs often experience significant access barriers to healthcare as their insurance program usually only reimburses health visits that take place in their hometown (11).

The National Health Commission in China (12) stated that OMWs, OMs for looking after offspring, and OMs for receiving care from family constituted the main three subgroups of OM. Researchers have used the China Migrant Dynamic Monitoring Survey to investigate the association between these three reasons for migration and healthcare use with this dataset (13), but they did not go far into the details of the differences among these three subgroups and the pathways of associated factors on healthcare use. Simultaneously, compare with other OMs, OMWs have the dual characteristics of the older population and migrant workers (14), which means they are more inherently disadvantaged and at higher risk of social inequities (13). Therefore, they may face greater challenges in access to health services. These barriers to accessing health services could adversely affect the wellbeing and health outcomes of OMWs (15, 16). In addition, although multiple studies have documented migrant workers’ barriers to accessing public services, there is scant research examining the inequalities in access to healthcare between OMWs and other OMs (17–19). Moreover, prior studies had not specified the paths of how the SHI registration place is associated with health services use across different OMs.

Therefore, this study aims to examine the differences in health services use among OMWs and other OMs (for looking after offspring and for receiving care from family) and explore the associated factors and paths of using health services use among subgroup OMs. Given that there has been an increase in migrants both within countries and internationally, targeted measures will shed light on the global community and inform policymakers who face similar challenges. Empirical strategies based on the differences among subgroups will contribute to identifying the vulnerable population and improving the accessibility of care delivery. The analyses of associated factors and paths offer insights to understand the relationship between health services use and the SHI registration place. Based on the above discussion, this study aims to answer two research questions as followed:

1) Are there differences in health services use between OMWs and other OMs?
2) What is the relationship between the registration place of SHI and health services use among OMWs and other OMs?

### Conceptual Framework

Anderson’s Behavioral model (ABM) focuses on explaining the individual and contextual determinants of healthcare utilization (20), which serves as a conceptual framework for our study to investigate the impact of SHI on healthcare use among OMs. Visiting doctors is suggested as the proxy for healthcare use to assess the availability of care delivery, illustrating whether the individual’s health needs are met (21). Andersen and Newman’s seminal model (20) proposed three major components of the determinants of healthcare use: predisposing, enabling, and need factors.

First, predisposing factors refer to the characteristics indicating the propensity for receiving healthcare, such as age and migration duration. Second, enabling factors refer to the conditions that enable individuals to seek healthcare (20). Health insurance is an essential enabling factor for health services use. Insurance coverage supplies financial resources which enable migrants to access health services and facilitate the receipt of care when needed (22). SHI in China plays a critical role in reducing financial barriers to accessing needed services and alleviating the economic burden of disease (22). The association between insurance and healthcare use may differ by the services coverage and co-payment. Rural-urban area of residence is another enabling factor critical for healthcare use highlighted by previous research (20). As noted in the Case Study, the insurance benefits differ across urban and urban health insurance programs. Additionally, health expenditures also indicate the individual’s capability to fend off the negative influences from other stressors on health status and the resources that enable individuals to seek healthcare (20). However, excessive expenditures would adversely affect health services use, especially for patients from low socioeconomic backgrounds (23). Insured older adults are less worried about health expenditure and have a higher receipt of care compared with the uninsured (24). Third, need factors, i.e., perceived and evaluated illness, capture the immediate cause of healthcare use (20). As individuals’ health status go worse, they are more likely to have greater health needs. Self-assessed health, which indicates the individual’s subjective assessment, is categorized as a need factor and used as the control variable in this study.

### Case Study of China

This study chooses China as the case study country as China has developed a population management scheme based on household registration and an established SHI scheme (25). Previous research has identified better employment opportunities as a key reason for migration when economic disparities in earnings and livelihoods manifest (26). Simultaneously, existing literature supports social and cultural motivations behind migrations, such as family ethics, health needs, and social network (27).

In China, prices and healthcare quality control do vary between OMWs and other OMs, but inequalities in benefits exist across OMs insured through the urban and rural health insurance schemes (6). The SHI in China includes the New Rural Cooperative Medical Scheme (NCMS), Urban Resident Insurance (URI), and Urban Employee Insurance (UEI). Altogether these schemes cover rural residents, urban employees, and urban residents who do not have formal employment (28). Since 2016, the Urban-Rural Resident Medical Insurance (URRMI) was implemented in stages by combing URI and NCMS (28); however, this did not change the fact that the urban health financing program, UEI, provides higher reimbursement rates than URRMI (6). Similarly, China carried out a new round of medical reform in 2019, which raised the government subsidy and services coverage for the insured and extend the coverage of the direct reimbursement for seeking cross-province inpatient services healthcare (8). However, the co-payment of inpatient services for UEI beneficiaries was still much lower than those enlisted in URRMI (24.4% vs. 40.3%) (9).

One crucial objective of this study is to examine whether visiting doctors during times of illness among OMs with SHI in their current place of residence is different from those without. SHI in the current place of residence here refers to two health insurance schemes in urban areas: URRMI registered in the urban area and UEI. The “territorial principle” has been the primary principle of population management policy in China—residents were under localized management in the registration area (29). SHI was also under localized management in the registration area. Although URRMI covers both rural and urban residents, the majority of rural-to-urban migrants enlisted in URRMI are registered in their hometowns—mostly rural areas (13). To enroll in URRMI in the current place of residence, they need to hold a non-agricultural *Hukou* (30). Furthermore, the UEI enrolment conditions mainly involve having formal employment and a non-agricultural *Hukou* (4). Even if these criteria have been met, rural-to-urban migrants need to go through a complex and time-consuming before enrolling in SHI in the current place of residence. Therefore, a large number of rural-to-urban OMs usually register in their place of origin rather than their current place of residence. A piece of research shows that, in China, a considerable proportion of OMs, especially for OMWs, are still covered by SHI registered in rural areas and benefit less than urban residents, which results in insufficient receipts of needed healthcare (29).

## Methods

### Data and Sample

Data are drawn from the 2015 wave of the China Migrant Dynamic Monitoring Survey (CMDMS) (31). CMDMS is a nationally representative interview survey of internal migrants in China, which. aims to investigate the health, healthcare, migration, and demographic information of people migrating within China (32, 33). This survey used the multi-stage stratified probability proportionate to size (13) method as the sampling technique (13), with a highly representative of national migrant data in China. Based on the PPS sampling technique, CMDMS selected communities and cities from 32 provincial-level units in China; then, this survey randomly recruited individuals in the selected communities (13). CMDMS has conducted the desk review and this sampling strategy was compiled based on the migrant statistics in the 2014 annual report of the China National Health Commission and the latest statistics of each provincial health commission (31).

The latest CMDMS specifically targeted OMs was merely conducted in 2015, and there is no alternative for nationally representative surveys on OMs in China. The 2015 CMDMS collected information on socio-demographic characteristics, health, and health services use among older people within migrating families. As noted before, the gap in coverage and benefits between rural and urban health insurance schemes still exists after the 2019 medical reform (9). Prior studies (34, 35) also used this 2015 dataset to investigate the relationship between health insurance, household registration, and health services use among OMs in China after the 2019 medical reform. In this regard, 2015 nationally representative CMDMS may be the most available dataset for research on Chinese OMs and well serves our aim of this study. Hence, we draw the data from the 2015 CMDMS dataset and the sample encompasses individuals who were aged above 60 years and migrated within the mainland of China. Guided by a report from the China National Health Commission (12), looking after offspring, seeking better employment opportunities, and to live close to their adult children constitute the main reasons for migration among older migrants. Prior research also investigated the healthcare use among Chinese OMs across these reasons for migration (13). In addition, as noted in the introduction, OMWs could be particularly vulnerable among the subgroups. Therefore, we divided the subjects into three categories: OMs for better employment opportunities (OMW, N = 3,050), OM for looking after children (N = 4,439), and OMs for receiving care from family (N = 4,184). Table 1 summarized the weighted statistics summary.

**TABLE 1:** Weighted descriptive statistics of older migrants (China. 2015).

Variables	OMWs	OMs for looking after offspring	OMs for receiving care from family
	Mean (SD)/Percentages		
Visiting doctors (%)	42.22%	53.58%	53.17%
SHI in current place of residence	9.99%	4.02%	7.15%
Age (years old)	63.62 (0.12)	65.30 (0.12)	70.18 (0.25)
Male (%)	72.11%	43.18%	48.47%
Highest educational level (%)			
No education	14.25%	17.74%	22.11%
Primary/secondary education	74.96%	65.3%	57.39%
High school or above	10.79%	16.96%	20.51%
MHEPA (CNY)	1040.30 (24.28)	1396.35 (24.44)	1274.72 (33.41)
Marital status (%)			
Unmarried	0.62%	0.05%	0.17%
Married	92.00%	81.53%	73.05%
Divorced/widowed	7.38%	18.42%	26.78%
Migration duration (years)	7.74 (0.22)	5.77 (0.13)	6.80 (0.17)
Self-assessed health (%)			
Healthy	63.08%	53.99%	37.53%
Generally healthy	32.37%	41.77%	45.88%
Unhealthy but can self-care	4.26%	4.13%	13.55%
Cannot self-care	0.29%	0.11%	3.04%
*N*	3,050	4,439	4,184

OMW, older migrant workers; OM, older migrants; SHI, social health insurance; SD, standard deviation; MHEPA, monthly household expenditure *per capita*. Percentages are shown for categorical variables, and Mean is shown for continuous variables. Migrant Dynamic Monitoring Survey, China, 2015.

### Variables

Based on existing literature and data availability (13, 32), the outcome of interest is visiting doctors. CMDMS collected information on this variable by asking: “How do you deal with the illness when you are sick.” This study aggregated this answer to create a dichotomized variable (visiting doctors): coded as 1 = yes (visiting doctors) and 0 = no (no medical treatment/self-treatment) (36). The key variable of interest is SHI in the current place of residence, a dichotomized variable coded as 1 = yes (SHI registered in the current place of residence), 0 = no (SHI registered in hometown/else areas).

Regarding the binary logistic regression analyses, this study controlled for a set of variables: age, gender, highest educational level, monthly household expenditure *per capita* (MHEPA), marriage, migration duration, and self-rated health status. Age is a categorical variable, coded as 1 = 60–70 years, 2 = 70–80 years, and 3 = 80 years or above (the reference group). Gender is a binary variable with “female” set as the reference category. The highest educational level is a categorial variable: no formal education (the reference group), primary or secondary education, and high school or above. Marriage has three categories: unmarried, married (the reference group), and divorced/widowed. MHEPA is a continuous variable, and we winsorized this variable in 0.5% quantile on both sides to rule out the impact of the extreme values and then logarithmically transformed it. Migrating duration is a continuous variable measured by years, winsorized in 0.5% quantile on both sides. Self-rated health is a categorical variable, coded as 1 = healthy, 2 = generally healthy, 3 = unhealthy but can self-care, and 4 = cannot self-care.

Based on the results of binary logistic models and existing literature (13, 32, 37), the measurement in path analysis models involves visiting doctors, age, monthly household expenditure *per capita* (MHEPA), migrating duration, and self-rated health status.

### Statistical Analysis

To explore the first research question, individual weights with non-respondent adjustment were applied for the sample statistics of visiting doctors among the three subgroups. To answer the second research question, firstly, this study employed binary logistic regression models to identify the associated factors of visiting doctors as the following equation:

$$y=logit\left(p\right)=\beta_{0}+\beta_{1}\times P_{m}+\sum_{m=7}^{m}\left(\beta_{m}\times C_{m}\right)$$

Where y refers to the probability model of the visiting doctors in a certain exposure of the independent factors. P_m_ denotes SHI in the current place of residence. C_m_ denotes control variables. β_0_ is the constant term. β_m_ are the coefficients of the respective regressors.

Based on the results of binary logistic regression analyses, significantly associated factors (SHI in current place of residence, age, MHEPA, migrating duration, and self-rated health status) were involved in the path analysis models. This study performed path analysis models for the three subgroup OMs. We first used path analysis to understand the relationship among the registration place of SHI, visiting doctors, and other variables among OMWs. The second part and the third part used the same path analysis to model the associations among OMs for looking after children and OMs for receiving care from family, respectively. The maximum likelihood method was used in the estimation. As shown in Table 1, the criteria to evaluate the model fitness were suggested by Deng Z (38): a value of χ^2^/df less than 3 and the root mean square error of approximation (RMSEA) less than 0.05 denote a well-fitted model for the data; the comparative fit index (CFI), normed fit index (NFI), Tucker-Lewis Coefficient (TLI), and incremental fit index over 0.90 show good fit indexes (38). Furthermore, to check the robustness of the result, a categorical variable, age, was replaced by a continuous variable measured by years in the three path analysis models.

## Results

Table 1 shows the weighed sample statistics of the three subgroup OMs. The rate of visiting doctors in OMWs (42.22%) was lower than that in OMs for looking after children (53.58%) and OMs for receiving care from family (53.17%). OMWs had a higher proportion of receiving formal education, longer migration duration, being male, married, and healthy than the other two subgroups, whereas OMWs had lower monthly household expenditure *per capita*.

Table 2 shows the results of binary logistic models to identify the factors associated with visiting doctors. Having SHI registered in the current place of residence is associated with increased odds of visiting doctors among OMWs and OMs for receiving care from family. In addition, greater monthly household expenditure *per capita* is related to an increased probability of doctor visits, whereas longer migration duration is associated with a decreased occurrence of this seeking health services behavior among OMWs. OMs who migrated to receive care from family and were unable to take care of themselves were less likely to visit doctors when they were sick than their healthy counterparts.

**TABLE 2:** Binary logistic regression investigating the relationship between social health insurance in registration place of residence and visiting doctors among older migrants (China. 2015).

Variables	OMWs	OMs for looking after children	OMs for receiving care from family
	OR ^P^ (SE)		
SHI in registration place of residence (Ref: SHI in hometown/other areas)			
current place of residence	1.76*(0.41)	0.77 (0.21)	1.48*(0.28)
Age (Ref: ≥80 years old)			
60–70	2.05 (1.92)	0.93 (0.52)	0.96 (0.22)
70–80	2.19 (2.15)	1.09 (0.61)	0.91 (0.20)
Gender (Ref: female)			
Male	0.88 (0.15)	1.11 (0.14)	1.09 (0.14)
Education (Ref: no formal education)			
Primary or secondary education	1.13 (0.25)	1.15 (0.25)	1.04 (0.23)
High school or above	2.84**(0.89)	1.07 (0.17)	1.00 (0.17)
MHEPA (CNY)	1.35*(0.20)	1.02 (0.12)	1.05 (0.14)
Marital status (Ref: married)			
Unmarried	0.44 (0.37)	—	5.86 (6.39)
Divorced/widowed	1.52 (0.40)	1.02 (0.18)	1.41*(0.22)
Migration duration (years)	0.97**(0.01)	1.01 (0.01)	0.98 (0.01)
Self-assessed health (Ref: healthy)			
Generally healthy	0.93 (0.15)	1.00 (0.13)	0.91 (0.13)
Unhealthy but can self-care	0.94 (0.32)	1.35 (0.41)	0.72 (0.15)
Cannot self-care	1.53 (1.68)	1.52 (1.64)	0.33*(1.15)
Constant	0.05*(0.08)	0.86 (0.89)	0.94 (1.93)
*N*	3,050	4,439	4,184

OMW, older migrant workers; OM, older migrants; SHI, social health insurance; SE, standard error; MHEPA, monthly household expenditure *per capita*. *p < 0.05, **p < 0.01, ***p < 0.001. Migrant Dynamic Monitoring Survey, China, 2015.

**FIGURE 1:**
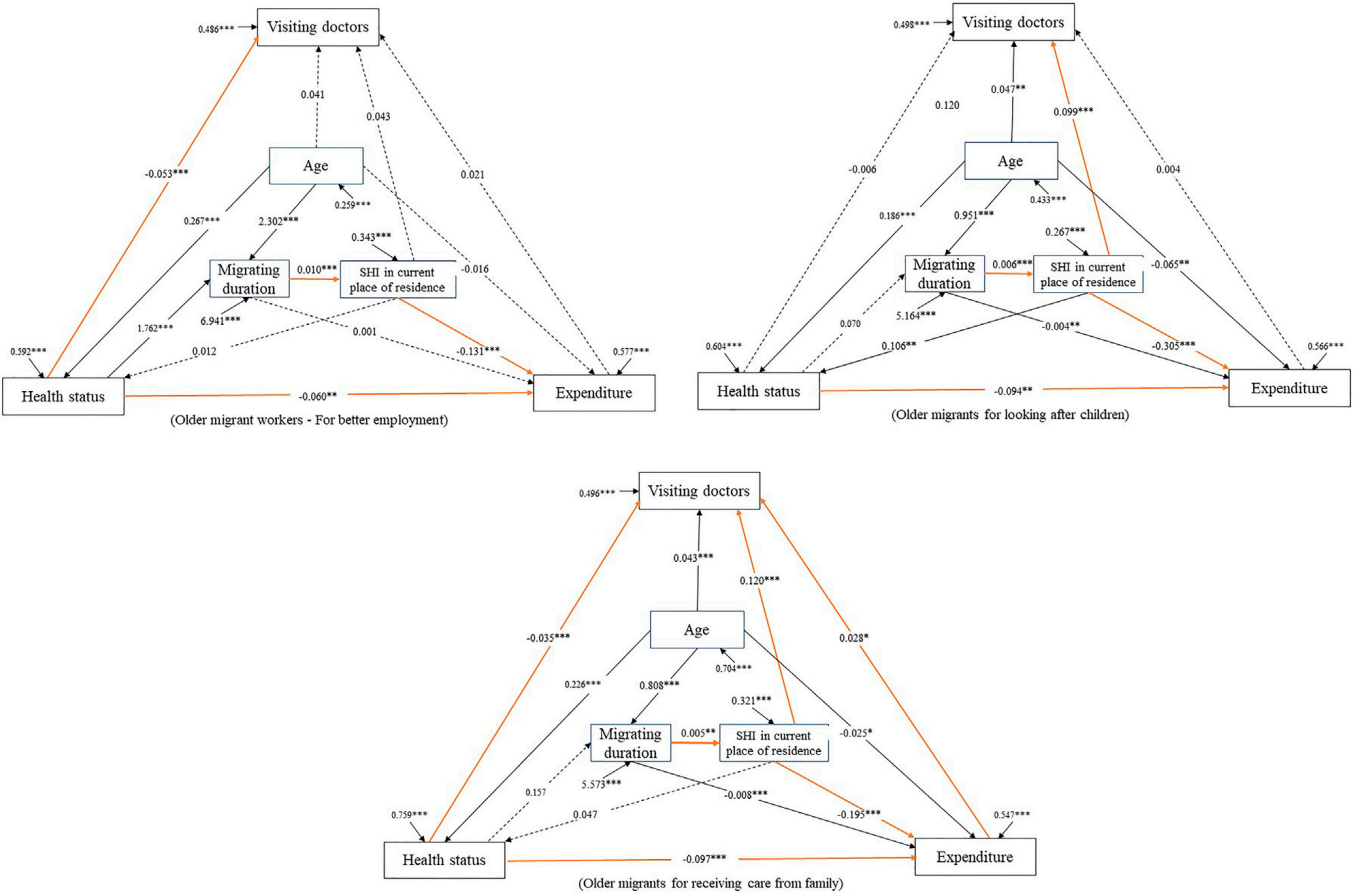
Path analysis models toward three subgroups of older migrants (China. 2015). SHI, social health insurance. *, *p* < 0.05; **, *p* < 0.01; ***, *p* < 0.001. Migrant Dynamic Monitoring Survey, China, 2015.

Table 3 reports the model fit of the path analysis models. All fit indices met the criteria, indicating acceptable model fitness between the hypothetic model and the data. Figure 1 shows the base case path analysis models for the three subgroups. Above all, a significant association of having SHI in the current place of residence with less household expenditure *per capita* was discovered in all three subgroup OMs. SHI in the current place of residence is significantly associated with increases in the occurrence of doctor visits among OMs for looking after children and OMs for receiving care from family, whereas this association is not observed in OMs for better employment. We also found a significant association between worse self-rated health and greater household expenditure *per capita* and an association between longer migration duration and having SHI in the current place of residence in all the subgroups. It is noted that worse self-assessed health was associated with a lower probability of visiting doctors among OMWs and OMs for receiving care from family. Additionally, regarding OMs for receiving care from family, household expenditure *per capita* demonstrated a positive association with visiting doctors.

**TABLE 3:** Summary of the fit indices (China. 2015).

Fit indices	χ^2^/df	RMSEA	CFI	NFI	TLI	IFI
Recommended value	<3	<0.08	>0.90	>0.90	>0.90	>0.90
Base case analysis						
OMWs	1.411	0.012	0.997	0.991	0.970	0.997
OMs for looking after children	1.234	0.007	0.999	0.993	0.985	0.999
OMs for receiving care from family	0.768	<0.001	1.000	0.997	1.011	1.001
Robustness check						
OMWs	1.561	0.014	0.996	0.991	0.963	0.997
OMs for looking after children	2.194	0.016	0.993	0.989	0.931	0.994
OMs for receiving care from family	0.933	<0.001	1.000	0.996	1.003	1.000

CFI, the comparative fit index; NFI, normed fit index; TLI, Tucker-Lewis Coefficient; IFI, incremental fit index; OMW, older migrant workers; OM, older migrants. Migrant Dynamic Monitoring Survey, China, 2015.

In the robustness check, this study changed the categorical variable, age, into a continuous variable measured by years. Table 4 compares the test statistics for the specific paths between the main models and robustness checks. The results of the robustness check, reported in the lower panel of Table 4, were in line with the results of the main models in the upper panel.

**TABLE 4:** Path coefficients in path analysis models among older migrants (China. 2015).

Base case analysis	OMWs	OMs for looking after children	OMs for receiving care from family
Path	Coefficients ^P^ (SE)		
Visiting doctors <- Health status	−0.053***(0.015)	−0.006 (0.012)	−0.035***(0.010)
Visiting doctors <- SHI in current place of residence	0.043 (0.027)	0.099***(0.030)	0.120***(0.026)
Visiting doctors <-Expenditure	0.021 (0.015)	0.004 (0.013)	0.028*(0.014)
Visiting doctors <- Age	0.041 (0.034)	0.047**(0.017)	0.043***(0.011)
Expenditure <- SHI in current place of residence	−0.131***(0.033)	−0.305***(0.035)	−0.195***(0.029)
Expenditure <- Health status	−0.060***(0.018)	−0.094***(0.001)	−0.097***(0.011)
Expenditure <- Migrating duration	0.001 (0.002)	−0.004**(0.002)	−0.008***(0.002)
Expenditure <- Age	0.016 (0.041)	−0.065**(0.020)	−0.025*(0.012)
SHI in current place of residence <- Migration duration	0.010***(0.001)	0.006***(0.001)	0.005***(0.001)
Health status <-SHI-in current place of residence	0.012 (0.035)	0.106**(0.036)	0.047 (0.039)
Health status <- Age	0.267***(0.041)	0.186***(0.001)	0.226***(0.017)
Migrating duration <- Age	2.302***(0.489)	0.951***(0.114)	0.808***(0.125)
Migrating duration <- Health status	1.762***(0.218)	0.070 (0.129)	0.157 (0.114)
Robustness check	OMWs	OMs for looking after children	OMs for receiving care from family
Visiting doctors <- Health status	−0.055*(0.015)	−0.007 (0.012)	−0.036***(0.010)
Visiting doctors <- SHI in current place of residence	0.042 (0.027)	0.098**(0.030)	0.120***(0.026)
Visiting doctors <-Expenditure	0.022 (0.015)	0.003 (0.013)	0.029*(0.014)
Visiting doctors <- Age	0.004 (0.003)	0.004**(0.002)	0.005***(0.001)
Expenditure <- SHI in current place of residence	−0.131***(0.033)	−0.304***(0.035)	−0.195***(0.029)
Expenditure <- Health status	−0.056**(0.018)	−0.095***(0.014)	−0.097***(0.011)
Expenditure <- Migrating duration	0.001 (0.002)	−0.004**(0.002)	−0.008***(0.002)
Expenditure <- Age	−0.004 (0.003)	−0.005*(0.002)	−0.002*(0.001)
SHI in current place of residence <- Migration duration	0.010***(0.001)	0.006***(0.001)	0.005***(0.001)
Health status <-SHI-in current place of residence	0.011 (0.023)	0.101**(0.036)	0.049 (0.039)
Health status <- Age	0.024***(0.004)	0.019***(0.002)	0.024***(0.002)
Migrating duration <- Age	0.219***(0.037)	0.112***(0.016)	0.079***(0.012)
Migrating duration <- Health status	1.704***(0.218)	0.033 (0.129)	0.140 (0.114)

OMW, older migrant workers; OM, older migrants; SE, standard error; SHI, social health insurance. *p < 0.05, **p < 0.01, ***p < 0.001. Migrant Dynamic Monitoring Survey, China, 2015.

## Discussion

This study offers four novel contributions to the existing research on older migrants. First, the analysis suggested that OMWs were the particularly vulnerable population in visiting doctors, and SHI, compared with other OMs. The results indicate that OMWs are more disadvantaged in using health services and socioeconomic status. OMWs had the least rate of visiting doctors in times of sickness, the least household expenditure, and the largest proportion of receiving secondary education or below among the three subgroups. Besides, given OMWs are still employed in their current place of residence after retirement to support their family, they are more likely to be from a low socioeconomic background and have unmet health needs. Previous research also evidenced the existence of significant unmet healthcare needs in Chinese rural-to-urban migrant workers (39).

Second, the negative relationship between worse health status and visiting doctors in OMs might be related to their disadvantages in low socioeconomic status. The path analyses demonstrate OMWs and OMs for receiving care from family who had worse health status are less likely to visit doctors, and worse health status was associated with less household expenditure. Disposable income and chronic disease might explain this finding. The CMDMS data indicate that the average monthly household disposable income *per capita* of OMWs and OMs for receiving care from family was merely CNY 1068 and CNY 1027 per month, while the figure was CNY 1363 among OMs for caring offspring. OMs for looking after children are more likely to live with their adult children who have formal employment and stable income resources. This is consistent with the broad body of literature which shows the role of income in using health services among older adults (40, 41). Simultaneously, the CMDMS data suggest that the prevalence of hypertension or diabetes in OMs for looking after offspring (24.6%) was higher than that of OMWs (12.5%). Due to the longstanding disease course, chronic diseases require regular healthcare and have more rigid demand, which is less influenced by socioeconomic status, and might incur increases in medical expenses. Research also shows that the price elasticity for older patients with high chronic diseases is higher than those without (42).

Third, a significant association between having SHI in the current place of residence and less expenditure was discovered in all three sub-group OMs. Moreover, regarding OMs for looking after offspring and OMs for receiving care from family, the expenditure reduced by the SHI in the current place of residence further increased the probability of visiting doctors. These findings might imply that addressing the inequalities in the reimbursement of health expenses among different types of SHI *via* unifying all types of health financing schemes into a single-payer program might be considered as a future approach. Due to the fragmented SHI schemes, rural and urban health insurance schemes are separately operated, and a gap exists in healthcare delivery between rural-to-urban migrants and urban residents (43). Although the Chinese government has implemented the Urban-Rural Resident Medical Insurance (URRMI) since 2016 by combing URI and NCMS (28), there are still 130 million Chinese people covered by NCMS in 2018 (6). On the one hand, since SHI is linked with the household registration scheme, a large number of rural-to-urban OMs is still covered by NCMS due to a lack of non-agricultural *Hukou* in urban area (29). Rural-to-urban OMs with SHI in their current place of residence benefit more from the reimbursement of healthcare expenses, in comparison to those without (4, 44). UEI which targets urban employees is evidence to be more effective in improving access to health services by providing higher financial protection, compared with other types of SHI (4). On the other hand, for those migrants who enrolled in URRMI, they still had a higher co-payment than urban employees enrolling in UEI (6). The high reimbursement of the urban health financing scheme could be a vital factor in the decision for OMs to seek health services (41). With a higher reduction in out-of-pocket health expenses benefiting from urban health financing schemes (45), OMs for receiving care from their family who have SHI in their current place of residence may be more likely to visit doctors during times of illness.

Fourth, OMWs with a longer migrating duration are more likely to have SHI in their current place of residence. This finding is in line with previous research that shows as the duration extends, rural-to-urban migrants are more likely to be eligible for urban health financing schemes (29). In the short run, rural-to-urban OMWs face policy barriers and discrimination in seeking employment and healthcare expenses reimbursement in their current place of residence. As time goes by, rural-to-urban migrants could undergo elaborate procedures in their current place of residence under transfer policies. OMWs might benefit from a long service for local employers (3, 46) to get enrolled in urban health financing schemes and other public welfare.

The following policy implications could be derived from the above analyses. First, policymakers are suggested to further promote the URRMI by combining the NCMS and UEI across rural residents and urban residents. Second, streamlined transfer policies for rural-to-urban migrants covered by NCMS to enroll in URRMI and improved elaborate procedures for OMWs to enroll in UEI are worthwhile. More efforts on community-based education or counseling on the transfer procedure on SHI in the current place of residence are needed. Third, we also suggest policymakers consider unifying all types of health insurance programs into a single-payer program to address the gap between existing URRMI and UEI. Fourth, the Chinese government has announced nationwide direct reimbursement for seeking cross-provincial inpatient expenses, but this reimbursement for outpatient expenses across provinces is still limited in pilot areas after the medical reform in 2019. We suggest the government to future extend the coverage of direct reimbursement for seeking cross-province outpatient services. Fifth, simply improving health services use among OMs *via* existing SHI is insufficient. We suggest policymakers in LMICs to consider a more generous public welfare scheme. Further programs targeting OMWs needed to be better designed to guarantee wage payment, pension, and other public benefits and improve access to health services. Sixth, to improve the awareness of active health-seeking behaviors during times of illness, social media and family members need to guide OMs to face their health needs and understand the importance of their health to the family.

### Limitations

The limitations need to be given due attention. First, given that the last national survey that investigated Chinese OMs was in 2015, the data merely reflected the status in 2015, and it could not explore the causal effects of cross-sectional data. Finally, although this study controlled for essential associated factors, restricted by method availability and the large sample size’s sensitivity, this study could not detect potential associations regarding other variables.

### Conclusion

OMWs are the particularly vulnerable population in using health services and socioeconomic status, in comparison to other OMs. OMWs and OMs for receiving care from their family who had worse health status were less likely to visit doctors during times of illness and were more likely to have less household expenditure, which might be related to the disadvantages in socioeconomic status and SHI. Having SHI in the current place of residence may help reduce household expenditure for OMs. As the migration duration extends, OMs are more likely to enroll in SHI registered in their current place of residence, which may increase the occurrence of doctor visits. Public policies in LMICs are worthy of consideration when more universal to cover OMWs. We urge the government to future promote URRMI across rural and urban areas. We suggest policymakers consider unifying all types of health financing schemes into a single-payer program by adjusting the approaches of existing URRMI and UEI. More efforts are needed to streamline transfer policies for rural-to-urban migrants to enroll in URRMI in the current place of residence or UEI.
